# Long-term xeno-free culture of human pluripotent stem cells on hydrogels with optimal elasticity

**DOI:** 10.1038/srep18136

**Published:** 2015-12-14

**Authors:** Akon Higuchi, Shih-Hsuan Kao, Qing-Dong Ling, Yen-Ming Chen, Hsing-Fen Li, Abdullah A. Alarfaj, Murugan A. Munusamy, Kadarkarai Murugan, Shih-Chang Chang, Hsin-Chung Lee, Shih-Tien Hsu, S. Suresh Kumar, Akihiro Umezawa

**Affiliations:** 1 Department of Chemical and Materials Engineering, National Central University, No. 300 Jhongli, Taoyuan, 32001 Taiwan; 2 Department of Reproduction, National Research Institute for Child Health and Development, 2-10-1 Okura, Setagaya-ku, Tokyo 157-8535, Japan; 3 Nano Medical Engineering Laboratory, RIKEN, 2-1, Hirosawa, Wako, Saitama 351-0198, Japan; 4 Department of Botany and Microbiology, College of Science, King Saud University, P.O. Box 2455, Riyadh 11451, Saudi Arabia; 5 Cathay Medical Research Institute, Cathay General Hospital, No. 32, Ln 160, Jian-Cheng Road, Hsi-Chi City, Taipei, 221, Taiwan; 6 Graduate Institute of Systems Biology and Bioinformatics, National Central University, No. 300, Jhongda RD., Jhongli, Taoyuan, 32001 Taiwan; 7 Division of Entomology, Department of Zoology, School of Life Sciences, Bharathiar University, Coimbatore, Tamil Nadu, India; 8 Department of Surgery, Cathay General Hospital, No.280, Sec. 4, Ren’ai Rd., Da’an Dist., Taipei, 10693, Taiwan; 9 Graduate Institute of Translational and Interdisciplinary Medicine, College of Health Science and Technology, National Central University, No. 300, Jhongda RD., Jhongli, Taoyuan, 32001 Taiwan; 10 Department of Internal Medicine, Taiwan Landseed Hospital, 77, Kuangtai Road, Pingjen City, Taoyuan 32405, Taiwan; 11 Department of Medical Microbiology and Parasitology, Universities Putra Malaysia, Serdang 43400, Slangor, Malaysia

## Abstract

The tentative clinical application of human pluripotent stem cells (hPSCs), such as human embryonic stem cells and human induced pluripotent stem cells, is restricted by the possibility of xenogenic contamination resulting from the use of mouse embryonic fibroblasts (MEFs) as a feeder layer. Therefore, we investigated hPSC cultures on biomaterials with different elasticities that were grafted with different nanosegments. We prepared dishes coated with polyvinylalcohol-co-itaconic acid hydrogels grafted with an oligopeptide derived from vitronectin (KGGPQVTRGDVFTMP) with elasticities ranging from 10.3 to 30.4 kPa storage moduli by controlling the crosslinking time. The hPSCs cultured on the stiffest substrates (30.4 kPa) tended to differentiate after five days of culture, whereas the hPSCs cultured on the optimal elastic substrates (25 kPa) maintained their pluripotency for over 20 passages under xeno-free conditions. These results indicate that cell culture matrices with optimal elasticity can maintain the pluripotency of hPSCs in culture.

Human pluripotent stem cells (hPSCs), such as human embryonic stem cells (hESCs)^1^ and induced pluripotent stem cells (hiPSCs)^2^,^3^, are a promising cell source for regenerative medicine, disease modeling, and drug screening because they can differentiate into specialized cells that originate from all three germ layers^4^,^5^,^6^. The development of a fully defined and xeno-free microenvironment for hPSC culture is necessary for the use of hPSCs in cell therapy and tissue engineering. The current gold standards for maintaining hPSCs in a pluripotent (undifferentiated) state are: (a) culture on feeder cells such as mouse embryonic fibroblasts (MEFs) or human fibroblasts and (b) culture on Matrigel or Geltrex^7^,^8^. Because Matrigel and Geltrex are extracted from the sarcomas of Engelbreth-Holm-Swarm mice, both gold standard hPSC culture systems are undefined; therefore their xenogenic components hinder the clinical application of hPSCs.

There is a need to develop cell biomaterials for feeder-free and xeno-free conditions for the expansion of hPSCs for clinical applications. Recently, several cell culture matrices that are chemically defined and devoid of xenogenic components have been developed for hPSC culture to maintain their pluripotency. The design of these cell culture matrices is based on the introduction of biological cues, such as extracellular matrices (ECMs)^9^,^10^,^11^,^12^,^13^,^14^,^15^,^16^,^17^,^18^,^19^,^20^,^21^,^22^,^23^,^24^, oligopeptides derived from ECMs^25^,^26^,^27^,^28^,^29^,^30^,^31^,^32^,^33^,^34^,^35^,^36^,^37^,^38^, and completely synthetic organic molecules^39^,^40^,^41^,^42^,^43^,^44^,^45^, on cell culture dishes.

Dishes coated with recombinant extracellular matrices (ECMs) such as recombinant vitronectin, laminin (laminin-511, laminin-521 and laminin-322), and fibronectin (CellStart) showed excellent performance for hPSC cultures in chemically defined and/or serum-containing media^9^,^10^,^11^,^12^,^13^,^14^,^15^,^16^,^17^,^18^,^19^,^20^,^21^,^22^,^23^,^24^. Dishes immobilized with oligopeptides derived from ECMs were also reported to maintain hPSC pluripotency in chemically defined medium^25^,^26^,^27^,^28^,^29^,^30^,^31^,^32^,^33^,^34^,^35^,^36^,^37^,^38^. Completely synthetic dishes, such as PMVE-alt-MA (poly(methyl vinyl ether-alt-maleic anhydride)^39^), PMEDSAH (poly[2-(methacryloyloxy](ethyldimethyl-(3-sulfopropyl)ammoniumhydroxide^40^,^41^,^42^,^43^), APMAAm (aminopropylmethacrylamide^44^) and copoly(AEtMA-co-DEAEA) (copoly[2-(acryloyloxyethyl)] trimethylammonium-co-2-(diethylamino)ethyl acrylate]^45^), have been also developed for hPSC culture in chemically defined medium, although there are no cell binding sites on the surfaces of the synthetic dishes. Therefore, it is necessary to evaluate the mechanism of pluripotent maintenance of hPSCs on synthetic dishes.

Increasing evidence suggests that both the physical cues (i.e., elasticity (stiffness)) and biological cues from the cell culture biomaterials direct stem cell fate during proliferation and maintain their pluripotency and differentiation^46^,^47^. Human mesenchymal stem cells (hMSCs) tend to efficiently differentiate into specific lineages of cells when they are grown on biomaterials with an elasticity similar to the tissue of interest^46^. Engler *et al.* demonstrated that hMSCs cultured on soft substrates (with elastic properties similar to brain tissue) in expansion media differentiated spontaneously into early lineages of neural cells, while hMSCs cultured on substrates with elastic moduli similar to muscle and bone tissue differentiated into early lineages of myocytes and osteoblasts, respectively^46^. These effects were explained by the ability of hMSCs to spread across biomaterials and form cytoskeletal stress fibers. Importantly, the effect of elasticity in cell culture biomaterials on the differentiation fate of hMSCs is restricted to the early stages of differentiation and does not direct the mature differentiation stages of hMSCs^47^,^48^. Furthermore, there are some contradictory reports^47^,^49^,^50^ for this well-known finding reported by Engler *et al.*^47^ However, the physical cues produced by biomaterials during the proliferation or differentiation of hPSCs into specific cell lineages should be an important contributing factor for the design of cell culture biomaterials for stem cell proliferation and differentiation^47^.

The microenvironment has also been reported to dictate the consequences of hPSCs^7^,^8^. Chowdhury *et al.* reported that mouse ESCs (mESCs) could maintain pluripotency when cultured in the absence of exogenous leukemia inhibitory factor (LIF) on soft substrates (0.6 kPa) that matched the intrinsic stiffness of mESCs, whereas mESCs did not maintain pluripotency in conventional stiff culture polystyrene dishes (12 GPa) coated with collagen type I or on hydrogels with much stiffer moduli^51^.

Several notable investigations addressed the effects of the elasticity of cell culture biomaterials on the pluripotency and differentiation fates of hMSCs and mESCs^46^,^47^,^48^,^51^. However, little is known about the effect of elasticity of the biomaterials on the pluripotency fate and proliferation of hESCs. This lack of evidence motivated us to investigate the effect of the elasticity of hydrogels grafted with biologically active nanosegments on hPSCs.

In this study, we developed synthetic hydrogels consisting of poly(vinyl alcohol-co-itaconic acid) (PVA-IA) grafted with oligopeptides derived from vitronectin (oligoVN) to evaluate the physical effect of substrate stiffness on the pluripotency and proliferation fates of hESCs and hiPSCs. These hydrogels were prepared with different elasticities by controlling the crosslinking intensity (time) with glutaraldehyde. The elasticity of the PVA-IA hydrogels could be varied using the same chemical structure as the polymeric main chain with different crosslinking intensities. OligoVN could be spontaneously grafted with the carboxylic acid group of PVA-IA via N-(3-dimethylaminopropyl)-N’-ethylcarbodiimide hydrochloride (EDC) and N-hydroxysuccinimide (NHS) chemistry in an aqueous solution. PVA-IA hydrogels grafted with or without oligoVN were transparent, thereby allowing the evaluation of the morphology of hSPCs cultured on the PVA-IA hydrogels using microscopy techniques similar to those employed using conventional cell culture dishes. The goal of this study is to investigate the optimal elasticity of PVA-IA hydrogels grafted with oligoVN for the expansion of hSPCs in xeno-free medium for a long period of time (at least 20 passages).

## Results

### Physical characterization of PVA-IA hydrogel dishes

**Figure 1:**
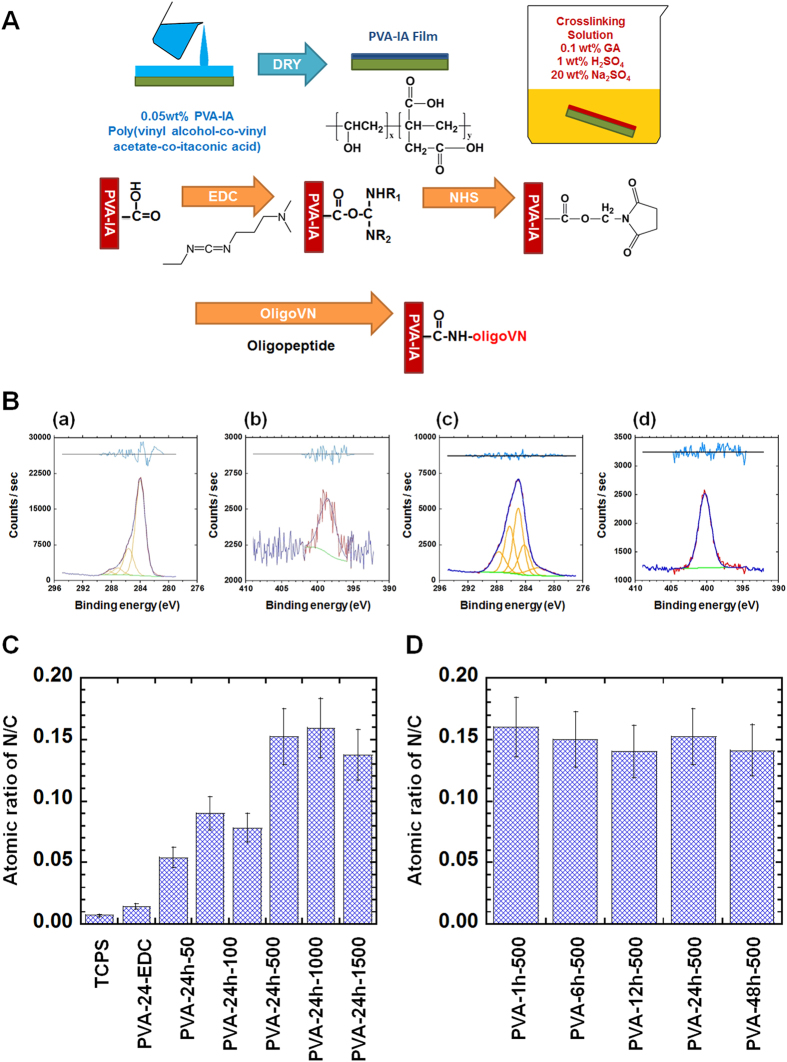
Preparation and characterization of PVA-IA hydrogels grafted with oligoVN. (**A**) Reaction scheme for PVA-IA hydrogels grafted with oligoVN. (**B**) High-resolution XPS spectra of the C1s (a and c) and N1s (**b** and **d**) peaks obtained on the surface of unmodified PVA-24h (a and b) and PVA-24h-1000 (c and d) dishes. (**C**) The atomic ratios of nitrogen to carbon (N/C) in TCPS, PVA-24h-EDC, and PVA-24h hydrogels grafted with different concentration of oligoVN (PVA-24h-50, PVA-24h-100, PVA-24h-500, PVA-24h-1000, and PVA-24h-1500). (**D**) The atomic ratios of nitrogen to carbon (N/C) in PVA-24h hydrogels grafted with different reaction time of oligoVN (PVA-1h-500, PVA-6h-500, PVA-12h-500, PVA-24h-500, and PVA-48h-1500).

**Figure 2:**
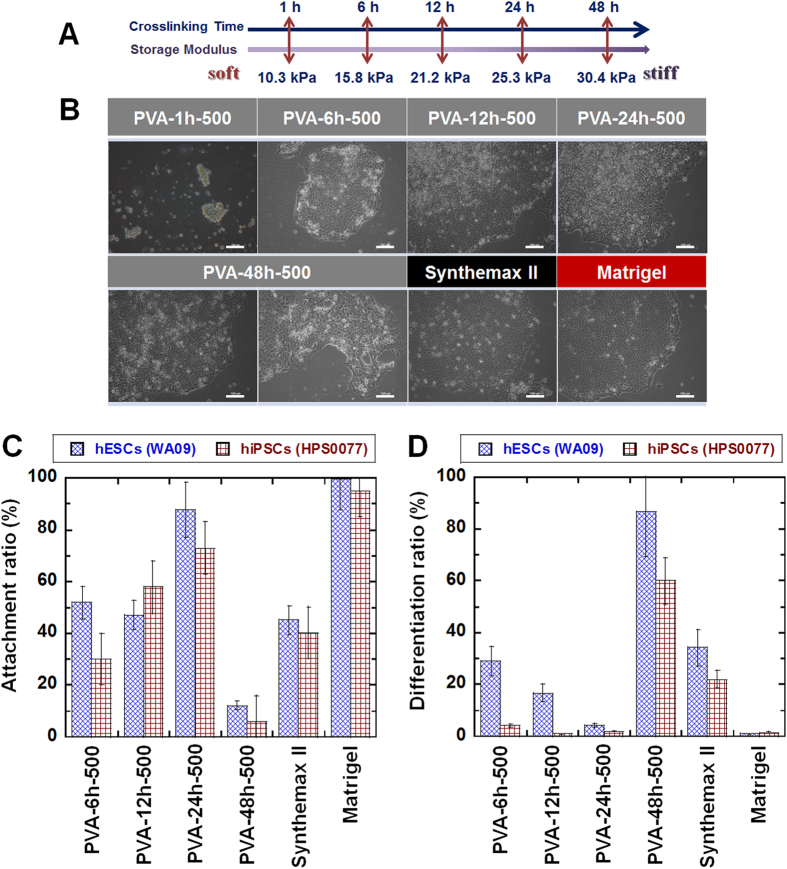
hPSC culture on PVA-oligoVN hydrogels with optimal elasticity. (**A**) Elasticity (storage modulus) is regulated by the crosslinking time on PVA-oligoVN hydrogels. (**B**) Morphology of hESCs (WA09) cultured on PVA-oligoVN hydrogels with several elasticities (PVA-1h-500, PVA-6h-500, PVA-12h-500, PVA-24h-500, and PVA-48h-500), Synthemax II dishes, and Matrigel at passage 1. The bar indicates 100 μm. (**C**) Attachment ratio of PSCs (blue bar, hESCs [WA09] and red bar, hiPSCs [HPS0077]) on PVA-oligoVN hydrogels with several elasticities, Synthemax II dishes, and Matrigel at passage 3. (**D**) Differentiation ratio of hPSCs (blue bar, hESCs [WA09] and red bar, hiPSCs [HPS0077]) on PVA-oligoVN hydrogels with several elasticities, Synthemax II dishes, and Matrigel at passage 3.

PVA-IA hydrogels grafted with oligoVN with different elasticities were prepared to investigate the optimal culture dish elasticity for hPSC expansion. The elasticity of PVA-IA hydrogels was controlled by the applied glutaraldehyde crosslinking intensity (time) (Fig. 1A). PVA-IA films with a 1.5 ± 0.3 μm thickness under dry conditions were used for these experiments. The thickness of the PVA-IA films (hydrogels) in the dishes in water was evaluated with a microgauge and determined to be 2.2–2.9 μm. The storage modulus *E*’ of self-standing PVA-IA hydrogels with a thickness of approximately 20–30 μm was also measured using a rheometer. The softest PVA-IA hydrogel included in this study (PVA-1h) had an *E*’ of 10.3 kPa, whereas the *E*’ of the hardest PVA-IA hydrogel in this study (PVA-48h) was 30.4 kPa, nearly 3-fold higher than the *E’* of PVA-1h (Fig. 2A). The *E’* of PVA-6h, PVA-12h, and PVA-24h hydrogels in this study was 15.8 kPa, 21.2 kPa, and 25.3 kPa, respectively (Fig. 2A). The storage moduli of PVA-IA hydrogels grafted with oligoVN were found to be approximately the same as those of the unmodified PVA-IA hydrogels within the range of experimental error, because the amount of oligoVN grafting was too small to contribute to the *E’* of the bulk PVA-IA hydrogels.

### Analysis of the chemical surface of PVA-IA dishes grafted with oligoVN

It is important to evaluate the existence of nanosegments (i.e., oligoVN) and the surface density of the nanosegments on PVA-IA hydrogels grafted with oligoVN that have different elasticities. However, determining the absolute quantity of grafted nanosegments on PVA-IA hydrogels via colorimetric (e.g., microBCA) or other chemical titration and reaction methods in this study was extremely difficult. Furthermore, the absolute quantity of nanosegments grafted onto the surface could not be evaluated using an ELISA (enzyme-linked immunosorbent assay) method. Therefore, we analyzed the surface of PVA-IA hydrogels grafted with or without oligoVN via XPS (X-ray photoelectron spectroscopy). Figure 1B provides the high-resolution XPS spectra of the C1s and N1s peaks on unmodified PVA-IA dishes (PVA-24h) and PVA-IA dishes grafted with oligoVN (PVA-24h-1000).

C–N bonding (285.9 eV), O–C = O bonding (289.3 eV), and C–C and C–H bonding (285.0 eV) were clearly observed in the XPS spectra obtained for the PVA-24h-1000 dishes (Fig. 1B(c)) compared to the PVA-24h dishes (Fig. 1B(a)). In contrast, C–C and C–H bonding (285.0 eV) were mainly observed in the XPS spectra of the PVA-24h dishes (Fig. 1B(a)). These findings suggest that oligoVN is covalently conjugated in the PVA-IA hydrogels.

The high-resolution XPS spectra of the N1s peaks obtained for the unmodified PVA-IA (PVA-24h, Fig. 2B(b)) and PVA-24h-1000 (Fig. 1B(d)) dishes were evaluated. An N1s peak at 399 eV^52^ was clearly observed in the PVA-24h-1000 dishes (Fig. 1B(d)). Conversely, only a faint N1s peak at 399 eV was found in the unmodified PVA-24h dishes (Fig. 1B(b)) due to the fact that PVA-IA does not initially include molecules containing nitrogen atoms; instead, nitrogen atoms can be derived from proteins and oligopeptides (i.e., oligoVN).

The atomic ratios of N/C on the PVA-24h dishes activated by EDC/NHS (PVA-24h-EDC) and the PVA-24h-oligoVN dishes were determined from the XPS spectra and shown in Fig. 1C. The N/C ratios of tissue culture polystyrene (TCPS, negative control) and PVA-24h-EDC were minimal. The N/C ratio increased with the increasing concentration of oligoVN used for grafting on the PVA-24h-oligoVN dishes up to 500 μg/ml, whereas the N/C ratio of PVA-24h-oligoVN reacted with concentrations of oligoVN ≥ 500 μg/ml were approximately the same within experimental error. This result indicates that the surface density of oligoVN grafted onto the PVA-IA hydrogels becomes saturated when the concentration of oligoVN is greater than 500 μg/ml.

The N/C ratios of PVA-IA hydrogels grafted with 500 μg/ml of oligoVN with different elasticities was also investigated (Fig. 1D). The N/C ratios of PVA-IA hydrogels grafted with the same concentration of oligoVN was approximately the same as PVA-IA hydrogels with different elasticities (i.e., PVA-1h-500, PVA-6h-500, PVA-12h-500, PVA-24h-500 and PVA-48h-500 dishes). This result is because the same concentration of oligoVN was used to graft onto the PVA-IA hydrogels. Thus, the surface density of oligoVN grafted onto PVA-IA hydrogels with different elasticities prepared with the same concentration of oligoVN is expected to be the same.

### hPSC culture on PVA-oligoVN hydrogels with optimal elasticity

hPSCs were cultured on PVA-oligoVN hydrogels with different elasticities to evaluate the effect of the elasticity of the hydrogels on the expansion of hPSCs and their ability to maintain their pluripotency. The first screening to evaluate biomaterials for hPSC culture is to evaluate the attachment of hPSCs and investigate the morphology of hPSC colonies showing no differentiation, because hPSCs cultured on adequate biomaterials show good attachment and colony morphology shapes that are characteristic of hPSCs. Figure 2B shows the morphology of hESCs (WA09) cultured on PVA-oligoVN dishes with several elasticities (PVA-1h-500, PVA-6h-500, PVA-12h-500, PVA-24h-500, and PVA-48h-500 dishes) and dishes coated with commercially available Synthemax II (Synthemax II dishes) at passage 1. hESCs did not attach well on the soft PVA-1h-500 dishes, whereas hESCs attached well on the PVA-IA dishes with elasticities greater than 15 kPa (i.e., the PVA-6h-500, PVA-12h-500, PVA-24h-500, and PVA-48h-500 dishes). This result indicates that the minimum elasticity of the biomaterials is necessary to allow the attachment of hESCs to the biomaterials.

The attachment ratio of hPSCs cultured on PVA-oligoVN hydrogels, Synthemax II dishes and Matrigel was evaluated by microscopy at each passage; the results at passage 3 are shown in Fig. 2C. A high attachment ratio of hESCs (WA09) and hiPSCs (HPS0077) was observed on Matrigel and PVA-24h-500 dishes, whereas middle and low attachment ratios of hPSCs were found on the PVA-6h-500, PVA-12h-500, PVA-48h-500, and Synthemax II dishes. This result indicates that PVA-oligoVN hydrogels with optimal elasticity (PVA-24h-500, 25.3 kPa) show the highest attachment ratio among the PVA-oligoVN hydrogels. Synthemax II dishes showed a lower attachment ratio compared to the PVA-24h-500 dishes (*p* < 0.05) and a similar attachment ratio compared to hPSCs on the PVA-12h-500 dishes (*p* > 0.05).

hPSC pluripotency can be evaluated based on colony morphology and live staining of alkali phosphatase. Supplementary Figure 1 shows the hESC morphology of completely differentiated cells (a), partially differentiated cells (b), and pluripotent cells (c). No alkali phosphatase activity was detected in the completely differentiated cells (a). In contrast, while the edge of the colony of the partially differentiated cells did not exhibit alkali phosphatase activity, alkali phosphatase activity was detected in the center of the colony (b). The pluripotent cells exhibited good colony morphology and alkali phosphatase activity in most cells (c). The differentiation ratio was evaluated for the whole hPSC colony on the dishes and calculated using the following equation:


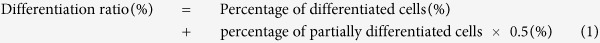


The differentiation ratios of hPSCs cultured on PVA-oligoVN hydrogels, Synthemax II dishes and Matrigel were evaluated based on microscopy at each passage; the results at passage 3 are shown in Fig. 2D. Low hPSC differentiation ratios were observed on the PVA-24h-500 dishes and Matrigel, whereas relatively high differentiation ratios were observed on the PVA-48h-500 and Synthemax II dishes. This result also suggests that the optimal elasticity of cell culture biomaterials is necessary to maintain pluripotency (e.g., 25.3 kPa) when hPSCs are cultured on PVA-oligoVN hydrogels. Based on the hPSC attachment and differentiation ratios, the optimal elasticity of PVA-oligoVN hydrogels was defined as 25.3 kPa in this study. A higher attachment ratio and higher pluripotency (lower differentiation ratio) of hPSCs were achieved on PVA-24h-500 dishes compared with the commercially available Synthemax II dishes (*p* < 0.05).

### Effect of oligoVN surface density on hPSC culture

**Figure 3:**
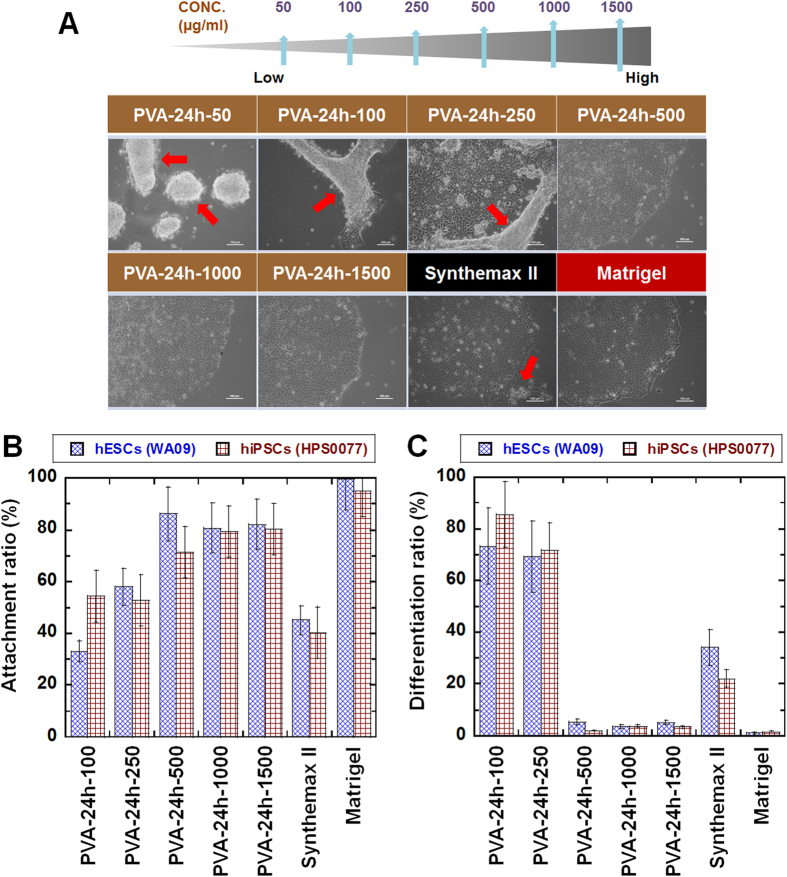
hPSC culture on PVA-oligoVN hydrogels with different surface densities of oligoVN. (**A**) Morphology of hESCs (WA09) cultured on PVA-oligoVN hydrogels with several surface densities of oligoVN (PVA-24h-50, PVA-24h-100, PVA-24h-250, PVA-24h-500, PVA-24h-1000, and PVA-24h-1500), Synthemax II dishes, and Matrigel at passage 1. The red arrows indicate detached cells. The bar indicates 100 μm. (**B**) Attachment ratio of hPSCs (blue bar, hESCs [WA09] and red bar, hiPSCs [HPS0077]) on PVA-oligoVN hydrogels with several surface densities of oligoVN, Synthemax II dishes, and Matrigel at passage 3. (**D**) Differentiation ratio of hPSCs (blue bar, hESCs [WA09] and red bar, hiPSCs [HPS0077]) on PVA-oligoVN hydrogels with several surface densities of oligoVN, Synthemax II dishes, and Matrigel at passage 3.

The optimal elasticity of PVA-oligoVN hydrogels for hPSC culture was evaluated to be 25.3 kPa (crosslinking time = 24 h) in the previous section. Next, the effect of the oligoVN surface density in PVA-oligoVN hydrogels on hPSC culture was investigated in the following experiments. The surface density of oligoVN was controlled by its concentration (50–1500 μg/ml) in the solution that was reacted with the PVA-24-EDC hydrogels. Figure 3A shows hESC (WA009) morphology after culture on PVA-oligoVN with variable oligoVN surface densities (PVA-24h-50, PVA-24h-100, PVA-24h-250, PVA-24h-500, PVA-24h-1000, and PVA-24h-1500 dishes), Synthemax II dishes and Matrigel at passage 1. hPSCs were found to detach easily from the PVA-oligoVN hydrogels prepared using oligoVN concentrations less than 500 μg/ml. Additionally, hPSCs could not be cultured on PVA-24h-50 dishes for more than two passages. The attachment ratio of hESCs (WA09) and hiPSCs (HPS0077) on each of the PVA-oligoVN hydrogels, Synthemax II dishes and Matrigel was evaluated at each passage until passage 10; the results at passage 3 are shown in Fig. 3B. The attachment ratio of hPSCs increased with increasing concentrations of oligoVN reacted with PVA-24h-EDC until the concentration reached 500 μg/ml. The attachment ratio of hPSCs on the PVA-24h-500 dishes was found to be higher compared to the PVA-24h-250 dishes (*p* < 0.05). The attachment ratio of hPSCs on the PVA-24h-500, PVA-24h-1000, and PVA-24h-1500 dishes was almost the same within the experimental error (*p* > 0.05). The attachment ratio of hPSCs on the PVA-24h-1500 dishes was found to be much higher than the Synthemax II dishes (*p* < 0.05) but slightly less than the Matrigel (*p* < 0.05).

To evaluate the maintenance of pluripotency of hPSCs cultured on PVA-oligoVN hydrogels, the differentiation ratios of hPSCs cultured on PVA-oligoVN hydrogels, Synthemax II dishes and Matrigel were evaluated using microscopy at each passage; the results at passage 3 are shown in Fig. 3C. An extremely low hPSC differentiation ratio was observed on the PVA-24h-500, PVA-24h-1000, and PVA-24h-1500 dishes and Matrigel, whereas a high differentiation ratio of hPSCs was observed on the PVA-24h-100 and PVA-24h-250 dishes and a relatively high differentiation ratio was found on the Synthemax II dishes. These results suggest that there is a threshold of oligoVN surface density (biological cues) on the PVA-oligoVN hydrogels that is needed to maintain hPSC pluripotency (i.e., low differentiation ratio). hPSCs attached to PVA-24h-oligoVN dishes prepared with more than 500 μg/ml of oligoVN and maintained their pluripotency to a level similar to that of cells cultured on Matrigel. It was necessary to use high concentration of oligoVN (500–1500 μg/ml) for the preparation of PVA-oligoVN hydrogels to maintain the pluripotency of hPSCs in this study. Active layer of Synthemax dishes is polyacrylate grafted with oligoVN and oligoVN used in Synthemax dishes is reported to be exactly the same sequence that we used for PVA-oligoVN hydrogels in this study^25^. High concentration of oligoVN such as 1 mM (1590 μg/ml) was reported to be used for the preparation of Synthemax dishes^25^. Therefore, it seems high concentration of oligoVN is necessary to keep pluripotency of hPSCs on the surface grafted with oligoVN.

### Long-term culture of hPSCs under xeno-free culture conditions

We found that the PVA-24h-500, PVA-24h-1000, and PVA-24h-1500 dishes were suitable cell culture biomaterials for hPSC culture by optimizing the elasticity (physical cues) and fine-tuning the oligoVN surface density (biological cues) in the previous sections. Then, long-term culture (20 passages) of hPSCs was evaluated on one of the optimized PVA-oligoVN hydrogels (PVA-24h-1000) and Synthemax II dishes using the xeno-free culture medium Essential 8 (xeno-free medium) and compared to hPSCs cultured on Matrigel.

**Figure 4:**
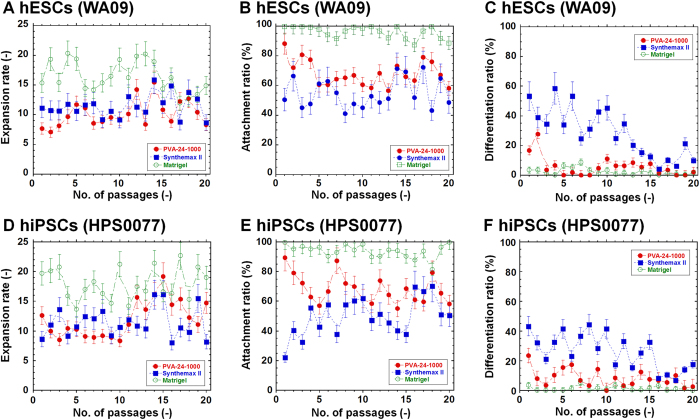
Long-term culture of hPSCs on PVA-oligoVN hydrogels with an optimal elasticity under xeno-free culture conditions. (**A**) Expansion rate of hESCs (WA09) on PVA-24h-1000 dishes (closed red circle), Synthemax II dishes (closed blue square), and Matrigel (open green circle) for 20 passages. (**B**) Attachment ratio of hESCs (WA09) on PVA-24h-1000 dishes (closed red circle), Synthemax II dishes (closed blue square), and Matrigel (open green circle) for 20 passages. (**C**) Differentiation ratio of hESCs (WA09) on PVA-24h-1000 dishes (closed red circle), Synthemax II dishes (closed blue square), and Matrigel (open green circle) for 20 passages. (**D**) Expansion rate of hiPSCs (HPS0077) on PVA-24h-1000 dishes (closed red circle), Synthemax II dishes (closed blue square), and Matrigel (open green circle) for 20 passages. (**E**) Attachment ratio of hiPSCs (HPS0077) on PVA-24h-1000 dishes (closed red circle), Synthemax II dishes (closed blue square), and Matrigel (open green circle) for 20 passages. (**F**) Differentiation ratio of hiPSCs (HPS0077) on PVA-24h-1000 dishes (closed red circle), Synthemax II dishes (closed blue square), and Matrigel (open green circle) for 20 passages.

Figure 4 shows the expansion rate, attachment ratio, and differentiation ratio of hESCs (WA09) and hiPSCs (HPS0077) cultured on PVA-24h-1000 dishes for 20 passages compared to those cultured on Synthemax II and Matrigel. The fold expansion of hESCs and hiPSCs on the PVA-24h-1000 dishes was found to be almost the same as the cells grown on commercially available Synthemax II dishes but was slightly less than cells grown on Matrigel (*p* < 0.05). The attachment ratio of hESCs and hiPSCs on the PVA-24h-1000 dishes was slightly higher compared to the Synthemax II dishes during 20 passages, but the difference did not reach statistical significance (*p* > 0.05). However, when the time of hPSC culture was limited to be only early passages (i.e., less than 5 passages), the attachment ratio of hESCs and hiPSCs on the PVA-24h-1000 dishes was found to be higher compared to the Synthemax II dishes with statistically significance (*p* < 0.05). The attachment ratio of hESCs and hiPSCs on Matrigel was always greater than 80% during the 20 hPSC culture passages, which was significantly higher compared to the PVA-24h-1000 and Synthemax II dishes (*p* < 0.05). The differentiation ratio of hESCs and hiPSCs on the PVA-24h-1000 dishes and Matrigel was much lower compared to Synthemax II (*p* < 0.05), indicating that hESCs and hiPSCs can maintain pluripotency on the PVA-24h-1000 dishes and Matrigel for a long period of time (i.e., at least 20 passages).

These results indicate that hPSCs can be cultured on PVA-24h-1000 dishes and Synthemax II under feeder-free and xeno-free conditions, although hPSC cultures on Matrigel exhibited a better expansion rate and attachment ratio. However, the hPSC culture on the Matrigels was performed under xeno-containing conditions, whereas the hPSC culture on the PVA-24h-1000 and Synthemax II dishes was performed under xeno-free conditions that were preferable for future clinical applications. Furthermore, hPSC culture on the PVA-24h-1000 dishes was preferable to culture on the commercially available Synthemax II, especially at a lower differentiation ratio. Thus, hPSCs maintain higher pluripotency on the PVA-24h-1000 dishes compared to the Synthemax II dishes. This finding was also verified in the following experiments.

**Figure 5:**
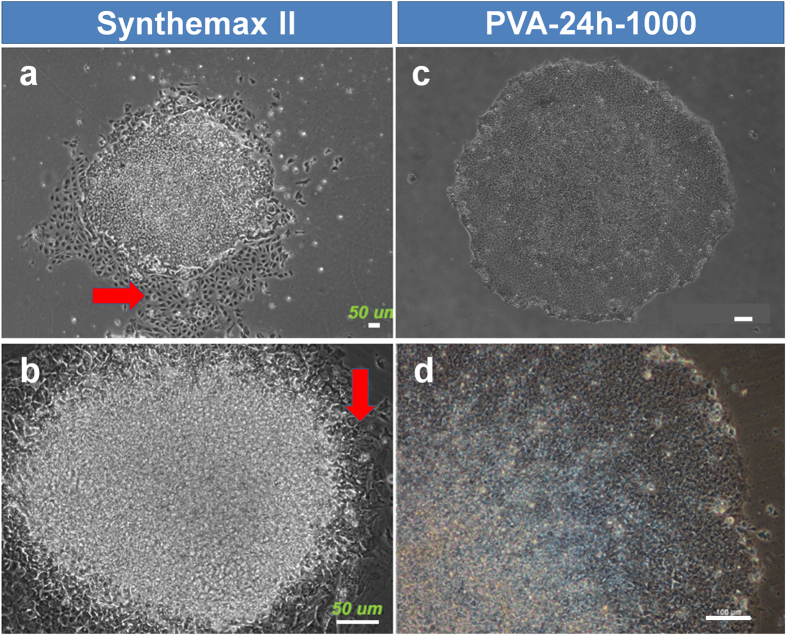
Comparison of hESC cultures on Synthemax II and PVA-oligoVN hydrogels. The morphology of hESCs (WA09) cultured on Synthemax II (**a,b**) and PVA-24h-1000 (**c,d**) dishes at passage 1 when hPSCs were shifted from culture on MEFs into culture on Synthemax II or PVA-24h-1000 dishes. Red arrows indicate differentiated hESCs. The bar indicates 50 μm (**a**,**b**) and 100 μm (**c,d**).

The results shown in Figs 2, 3, 4 were obtained using hPSCs cultured on Matrigel for nine passages in advance. This process is important for hPSCs to become accustomed to feeder-free conditions. We also performed hPSC cultures directly on Synthemax II and PVA-24-1000 dishes using hPSCs cultured on MEFs that were not cultured on Matrigel in advance; the results are shown in Fig. 5. hPSCs shifted directly to the Synthemax II dishes were found to more easily differentiate at passage one, whereas hPSCs could maintain pluripotency following direct transfer to the PVA-24h-1000 dishes. These results are consistent with those shown in Fig. 4C,F where the differentiation of hPSCs was more predominant on the Synthemax II dishes than on PVA-24h-1000 dishes.

**Figure 6:**
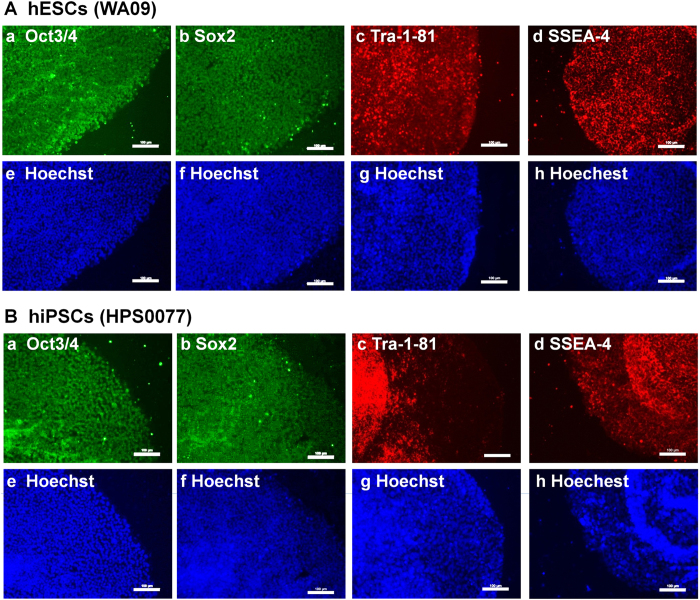
Characterization of pluripotency of hPSCs (hESCs and hiPSCs) cultured on PVA-oligoVN hydrogels based on expression of pluripotent proteins. (**A**) Pluripotent protein expression on hESCs (WA09) analyzed by immunostaining after culture on PVA-24h-1000 dishes under xeno-free conditions for 20 passages. (**a**) Oct3/4, (**b**) Sox2, (**c**) Tra-1-81, (**d**) SSEA-4 and (**e–h**) Hoechest staining of hESCs used in (**a–d**). The bar indicates 100 μm. (**B**) Pluripotent protein expression on hiPSCs (HPS0077) analyzed by immunostaining after culture on PVA-24h-1000 dishes under xeno-free conditions for 20 passages. (**a**) Oct3/4, (**b**) Sox2, (**c**) Tra-1-81, (**d**) SSEA-4 and (**e–h**) Hoechest staining of hESCs used in (**a–d**). The bar indicates 100 μm.

hPSC pluripotency was evaluated based on the expression of pluripotent proteins on hESCs (WA09) and hiPSCs (HPS0077) by immunostaining after culturing on PVA-24h-1000 dishes for 20 passages; the results are shown in Fig. 6. The pluripotent proteins Oct3/4, Sox2, Tra-1-81, and SSEA-4 were expressed on hESCs and hiPSCs cultured on PVA-24h-1000 dishes under xeno-free conditions (i.e., in Essential 8 culture medium) for 20 passages.

### Differentiation ability of hPSCs *in vivo* and *in vitro*

**Figure 7:**
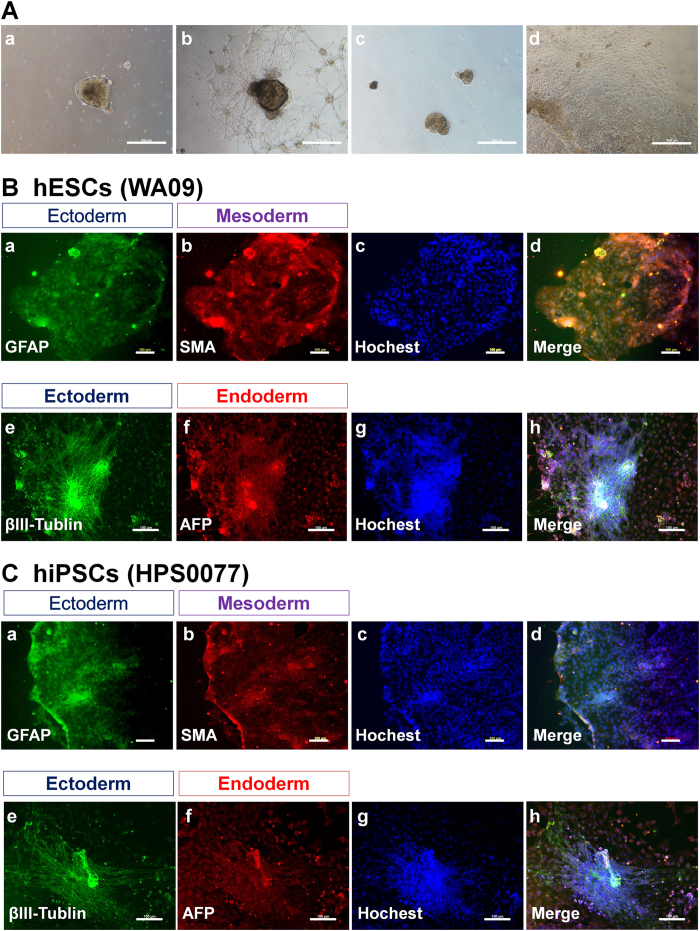
Characterization of the differentiation ability of hPSCs (hESCs and hiPSCs) *in vitro* after culture on PVA-oligoVN hydrogels for 20 passages. (**A**) Morphology of EBs differentiated from hESCs (WA09, **a,b**) and hiPSCs (HPS0077, **c,d**) after culture on PVA-24h-1000 dishes under xeno-free conditions for 20 passages. (**B**) Immunostaining of an ectoderm protein (**a**, GFAP; **e**, βIII-tubulin), mesoderm protein (**b**, SMA), and endoderm (**f**, AFP) protein on hESCs (WA09) after culture on PVA-24h-1000 dishes under xeno-free conditions for 20 passages. (**c**) Hoechest staining of hESCs used in (**a,b**). (**d**) Merged picture of (**a–c**). (**g**) Hoechest staining of hESCs used in (**e**,**f**). (h) Merged picture of (**e–g**). The bar indicates 100 μm. (**C**) Immunostaining of an ectoderm protein (**a**, GFAP; **e**, βIII-tubulin), mesoderm protein (**b**, SMA), and endoderm (**f**, AFP) protein on hiPSCs (HPS0077) after culture on PVA-24h-1000 dishes under xeno-free conditions for 20 passages. (**c**) Hoechest staining of hESCs used in (**a,b**). (**d**) Merged picture of (**a–c**). (**g**) Hoechest staining of hESCs used in (**e**,**f**). (**h**) Merged picture of (**e–g**). The bar indicates 100 μm.

It is necessary to evaluate whether hPSCs can differentiate into cells derived from all three germ layers *in vitro* (EB formation assay) and *in vivo* (teratoma formation assay) to evaluate their pluripotency. hESCs (WA09) and hiPSCs (HPS0099) were cultured on PVA-24h-1000 dishes under xeno-free conditions for 20 passages and subsequently cultured in suspension using ultra low protein binding dishes to form EBs (Fig. 7A). Differentiated hESCs and hiPSCs were immunostained with AFP (alpha-fetoprotein, endoderm), SMA (smooth muscle actin, mesoderm), βIII-tubulin (ectoderm), and GFAP (glial fibrillary acidic protein, ectoderm); the results are shown in Fig. 7B for hESCs and Fig. 7C for hiPSCs. Both hESCs and hiPSCs were able to differentiate into cells expressing AFP, SMA, βIII-tubulin, and GFAP, indicating that the hESCs and hiPSCs could maintain their pluripotency after culture on PVA-24h-1000 dishes under xeno-free conditions and differentiate into cells derived from the three germ layers *in vitro*.

**Figure 8:**
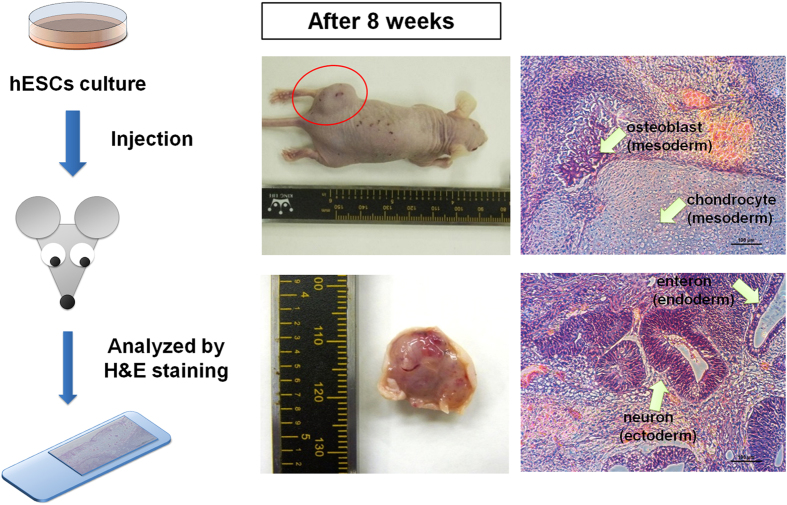
Characterization of the differentiation ability of hPSCs (hESCs and hiPSCs) *in vivo* after culture on PVA-oligoVN hydrogels for 20 passages. Pluripotency of teratoma-forming hESCs (WA09) after culture on PVA-24h-1000 dishes under xeno-free conditions for 20 passages. Osteoblasts and chondrocytes (mesoderm), neurons (ectoderm), and enterons (endoderm) can be detected. The bar indicates 100 μm.

We evaluated the ability of hESCs to differentiate into cells derived from the three germ layers *in vivo* using the teratoma formation assay. hESCs cultured on PVA-24h-1000 dishes for ten passages were subcutaneously xenotransplanted into non-obese diabetic/severe combined immunodeficiency (SCID) mice to generate teratomas (Fig. 8). Staining the teratomas with hematoxylin and eosin (H&E) demonstrated the presence of cells derived from the three germ layers (enteron (endoderm), osteoblasts (mesoderm), chondrocytes (mesoderm), and neuron (ectoderm)). These results suggest that hPSCs cultured long-term on PVA-24h-1000 dishes (10–20 passages) can maintain their pluripotency and are able to differentiate into cells derived from the three germ layers *in vitro* and *in vivo*.

## Discussion

Several hPSC culture substrates have been reported in feeder-free and chemically defined conditions. Some examples of hPSC culture substrates are briefly summarized in Table 1. Substrates coated or grafted with specific ECMs (fibronectin, laminin-511, laminin-521, laminin-332, and vitronectin)^9^,^10^,^11^,^12^,^13^,^14^,^15^,^16^,^17^,^18^,^19^,^20^,^21^,^22^,^23^,^24^ and oligopeptides^25^,^26^,^27^,^28^,^29^,^30^,^31^,^32^,^33^,^34^,^35^,^36^,^37^,^38^ derived from ECMs that have cell binding domains are typically used. Because the cost of production of ECMs originating from humans prepared under xeno-free conditions for good manufacturing practice (GMP) approval is high, hPSC culture substrates that immobilize ECM-derived oligopeptides represents a promising approach. Recently, completely synthetic polymers have been developed for use as hPSC culture substrates. For example, hPSCs can be cultured on APMAAm (aminopropylmethacrylamide), PMEDSAH (poly[2-(methacryloyloxy(ethyl dimethyl-(3-sulfopropyl)ammoniumhydroxide), and PMVE-alt-MA (poly[methyl vinyl ether-alt-maleic anhydride]) in chemically defined media^39^,^40^,^41^,^42^,^43^,^44^,^45^. However, hPSCs are cultured on Matrigel prior to culture on completely synthetic polymeric substrates, and Matrigel are derived under xeno-containing conditions. Furthermore, the mechanism of hPSC attachment to the completely synthetic polymer substrates is currently unknown. No systematic explanation for the molecular design of the synthetic polymer substrates based on hPSC attachment and proliferation has been reported. The other drawback of the use of completely synthetic polymeric substrates is that only polymer scientists and organic chemists can synthesize these types of complicated polymers, whereas ECMs and oligopeptides can be obtained commercially.

Currently, only coating materials and substrates are commercially available for hPSC culture substrates; these materials are composed of ECM-derived oligopeptides (with the exception of ECMs and chimeric proteins)^53^,^54^. No completely synthetic polymer substrates are commercially available for hPSC culture. Therefore, it is necessary to investigate whether hPSCs can be cultured on completely synthetic polymer substrates with high reproducibility by different groups of researchers if these substrates will be used for hPSC cultures in the future.

The PVA-24h-1000 dishes developed in this study contained the hPSC binding domain of oligoVN, which binds to integrins α_V_β_3_ and α_V_β_5_^8^. Therefore, hESCs cultured on MEFs can be directly shifted onto PVA-24h-1000 dishes without any differentiation, whereas hESCs cultured on Synthemax II dishes exhibit significant differentiation (Fig. 5), although the binding site of hPSCs on Synthemax II dishes is reported to be the same amino acid sequence to oligoVN used in PVA-oligoVN dishes in this study^25^.

The effect of the elasticity of cell culture matrices on stem cell fate in terms of pluripotency and differentiation has been primarily investigated for adult stem cells, such as mesenchymal stem cells and hematopoietic stem cells^46^,^47^,^48^. However, in this study we found that fine-tuning the elasticity of hPSC culture matrices was very important for the attachment and expansion of hPSCs. Only a few small or no hPSC colonies were observed on the softest substrates (10–20 kPa), whereas hPSCs cultured on the stiffest substrates (e.g., storage modulus of 30 kPa) tended to differentiate after five days of culture. hPSCs cultured on the optimal elastic substrates (25 kPa) maintained their pluripotency for more than 20 passages under xeno-free and feeder-free conditions. The culture of hSPCs on Synthemax II dishes for 20 passages under xeno-free and feeder-free conditions has been reported in the literature^25^. However, hPSCs tended to more easily differentiate on the Synthemax II dishes compared to hPSCs cultured on Matrigel and the PVA-24h-1000 dishes developed in this study. This result is because the base cell culture dishes of Synthemax II are stiff TCPS with an approximately 12 GPa modulus^48^, which is too stiff a surface for hPSC culture. If Synthemax II is coated on the softer hydrogels, the differentiation ability of hPSCs might decrease and the Synthemax II dishes would be more adequate for hPSC culture.

Matrigel-coated dishes supported long-term expansion of hPSCs maintaining their pluripotency. Matrigels are known to contain variety of ECMs as well as growth factors, which support long-term culture of hPSCs maintaing their pluripotency. Although Matrigels were coated on stiff TCPS, matrigel layer on TCPS may contribute to generate somewhat soft cell culture biomaterials (less stiffness compared to solely TCPS surface) for hPSCs that can be cultured for long-term and keep their pluripotency on Matrigel-coated dishes.

Musah *et al.* cultured hESCs on glycosaminoglycan-binding acrylamide hydrogels with 0.7 kPa, 3 kPa, and 10 kPa^33^. They found only relatively stiff hydrogels (10 kPa) could maintain hESC proliferation, which is similar to our results demonstrating that extremely soft hydrogels (0.7–3 kPa) cannot attach and maintain hESCs. Unfortunately, this study did not report hESC proliferation on hydrogels with a surface stiffer than 10 kPa, because the present study found that the optimal elasticity of the hydrogels was 25 kPa. However, their study indicates that hESCs can respond to mechanical information from cell culture matrices via glycosaminoglycan engagement, which can be fine-tuned to activate specific signaling pathways linked to pluripotency.

Chang *et al.* developed hydrogels containing heparin-mimicking moieties, which have different bulk modulus by varing their crosslinking density (54, 138, and 344 kPa)^55^. Their hydrogels with low bulk elasticity (54 kPa) supported minimal cell adhesion of hESCs and those having a moderate elasticity of 138 kPa demonstrated some cell adhesion of hESCs, but the attached cells exhibited spontaneous differentiation. They found the most rigid hydrogels in their study (344 kPa) exhibited good attachment of hESCs and supported long-term expansion of hESCs maintaining their pluripotency for >20 passages. Their hydrogels do not have direct binding site of hESCs, but have FGF-2 binding site due to the existence of heparin-mimicking moieties, which contribute to the binding of hESCs. Especially, their softer hydrogels have less binding of FGF-2 due to lower density of heparin-mimicking moieties. Therefore, their hydrogel needs minimum rigidity to attach hESCs, which contributes to the existence of the threshold amount of FGF-2 binding site (hESCs binding site) in their hydrogels. In our study, PVA-24h-1000 hydrogels having 25 kPa storage modulus are the best biomaterials to support long-term expansion of hPSCs maintaining their pluripotency. Both Chang’s work and present study demonstrated that there is the optimal elasticity of the hydrogels that can support long-term expansion of hPSCs. The difference of optimal elasticity among Chang’s work and present study to support long-term expansion of hESCs should be originated from different design of base materials of hydrogels.

Important considerations for the design of biomaterials for hPSC cultures include the optimization of the elasticity (physical cues) of the biomaterials and the selection and fine-tuning of the surface density of bioactive cell-binding nanosegments (biological cues) on the surface of the biomaterials. PVA-IA hydrogels with an optimal elasticity of 25 kPa and oligoVN concentrations greater than 500 μg/ml were selected in this study for optimal hPSC culture. The PVA-24h-500, PVA-24h-1000, and PVA-24h-1500 dishes showed higher attachment ratios and lower differentiation ratios of hPSCs compared to the commercially available Synthemax II dishes. This is because Synthemax II dishes are prepared by coating a bioactive polymer on conventional stiff tissue culture dishes; therefore, Synthemax II dishes do not have the optimal elasticity for the culture of hPSCs, whereas the PVA-oligoVN dishes developed in this study have optimized elasticity for hPSC culture due to the control of the crosslinking density of the PVA-IA hydrogels. hPSCs can be cultured on PVA-24h-1000 dishes for long-term passage (i.e., 20 passages) by maintaining their pluripotency and ability to differentiate into cells derived from the three germ layers both *in vitro* and *in vivo*.

## Conclusion

We have developed PVA-oligoVN hydrogels having different elasticity for long-term expansion of hPSCs maintaining their pluripotency for 20 passages. The optimal elasticity of cell culture biomaterials is necessary to maintain pluripotency (e.g., 25.3 kPa) when hPSCs are cultured on PVA-oligoVN hydrogels. It is also necessary to use high concentration of oligoVN (500–1500 μg/ml) for the preparation of PVA-oligoVN hydrogels to maintain the pluripotency. The optimization of the elasticity (physical cues) of the biomaterials as well as the selection and fine-tuning of the surface density of bioactive cell-binding nanosegments (biological cues) on the surface of the biomaterials is important for the design of biomaterials for long-term expansion of hPSCs maintaining their pluripotency.

## Materials and methods

### Materials

hESCs (WA09) were obtained from the WiCell Research Institute, Inc (Madison, WI, USA). hiPSCs (HPS0077) were obtained from the Riken BioResource Center (Tsukuba, Japan). Corning Synthemax II-SC (3535XX1) was purchased from Sigma-Aldrich (St. Louis, MO, USA). Matrigel (354230, growth factor-reduced basement membrane matrix) was obtained from BD Biosciences (San Jose, CA, USA). mTeSR1 medium (05850) was obtained from Stem Cell Technologies. Essential 8 medium (A1517001), Essential 6 medium (A1516401), DMEM/F12 medium (11330-057), and KnockOut Serum Replacement (10828-028) were purchased from Life Technology. The oligopeptide of oligoVN (KGGPQVTRGDVFTMP) was obtained from PHJapan (Hiroshima, Japan). TCPS dishes (diameter = 35 mm, 35-3001) were purchased from Becton Dickinson (Franklin Lakes, NJ, USA). N-(3-Dimethylaminopropyl)-N’-ethylcarbodiimide hydrochloride (EDC, 03450), N-hydroxysuccinimide (NHS, 13062), and glutaraldehyde (25% in water, G5882) were obtained from Sigma-Aldrich (St. Louis, MO, USA). The antibody against Oct3/4 (anti-octamer-binding transcription factor 3/4 IgG, sc-5279, mouse) was obtained from Santa Cruz Biotechnology (Dallas, TX, USA). The antibody against Sox2 (anti-SRY (sex determining region Y)-box 2 IgG, ab5603, rabbit) and the alkaliphosphatase detection kit (SCR004) were obtained from EMD Millipore (Billerica, MA, USA). The antibodies against SSEA-4 (anti-stage-specific embryonic antigen-4 IgG, ab16287, mouse) and TRA-1-81 (anti-tumor rejection antigen-1-81 IgG, ab16289, mouse) were purchased from Abcam (Cambridge, MA, USA). The antibodies against GFAP (anti-glial fibrillary acidic protein IgG, MA5-15086, mouse), AFP (anti-alpha-fetoprotein IgG, PA5-21004, mouse), SMA (anti-smooth muscle actin IgG, PA5-19465, rabbit), Alexa Fluor 488 goat anti-mouse IgG (H + L) (A11001), Alexa Fluor 488 goat anti-rabbit IgG (H + L) (A11008), and Alexa Fluor 594 donkey anti-rabbit IgG (H + L) (A21207) were purchased from Life Technology (Carlsbad, CA, USA). The antibody against βIII-Tubulin (MCA2047, mouse) was obtained from AbD Serotec (Raleigh, NC, USA). Hoechst 33342 (PA-3014) was obtained from Lonza (Basel, Switzerland). The other chemicals employed were reagent grade and were used without further purification; these chemicals were purchased from Sigma-Aldrich (St. Louis, MO, USA). Ultrapure water produced by a Milli-Q system (Millipore Corporation, Billerica, MA, USA) was used throughout the experiments.

### Preparation of crosslinked PVA-IA hydrogel dishes

PVA-IA (Japan VAM & Poval Co., Ltd., Osaka, Japan) with 1.3 mol% itaconic acid showing a degree of hydrolysis of 97.7% and a degree of polymerization of 1750 was dissolved to 0.05 wt% for the cell culture experiments or 0.5 wt% for the rheometer measurements in ultrapure water. Then, the solutions were agitated for two days and subsequently kept at room temperature for one day to ensure that no air bubbles were present^48^. A 3 mL aliquot of the PVA-IA solution was added to a 35 mm TCPS dish and dried for a week on a clean bench to produce a film. The PVA-IA films were immersed in an aqueous crosslinking solution composed of 1 wt% glutaraldehyde, 20 wt% sodium sulfate, and 1 wt% sulfuric acid for 0.5, 1, 6, 24, and 48 h (Fig. 1). The naming convention ‘PVA-*X*’ (e.g., PVA-24h) indicates PVA-IA hydrogels crosslinked for *X* hours (e.g., 24 h). After crosslinking, the PVA-IA hydrogels were washed with ultrapure water and then immersed in ultrapure water. The ultrapure water was changed twice daily prior to grafting with oligoVN and use in cell culture. The PVA-IA hydrogels were sterilized via immersion in a 75 v/v% ethanol solution overnight, washed in ultrapure water and kept in ultrapure water until use for cell culture.

### Preparation of PVA-IA hydrogel dishes grafted with oligoVN

Following the preparation of PVA-IA hydrogels with different elasticities, the hydrogels were activated via immersion in an aqueous solution containing 10 mg/ml EDC and 10 mg/ml NHS for 6 h at 25 °C. Subsequently, the PVA-IA hydrogels were washed with phosphate buffered saline (PBS, pH7.2) and immersed in a PBS solution containing 100–1500 μg/mL of oligoVN for 24 h at 4 °C (Fig. 1). After grafting oligoVN, the PVA-IA hydrogels were washed with ultrapure water for 12 h to remove residual oligoVN. The PVA-IA hydrogels grafted with *Y* μg/mL of oligoVN are hereafter referred to as PVA-*X*h-*Y*, where *X* indicates the crosslinking time (h).

### Characterization of PVA-IA hydrogel dishes grafted with oligoVN

The chemical composition of the surface-grafted PVA-IA hydrogel dishes was measured using X-ray photoelectron spectroscopy (XPS, K-Alpha spectrometer, Thermal Scientific, Inc., Amarillo, TX, USA, equipped with a monochromatic Al-K X-ray source [1,486.6 eV photons]). The energy of the emitted electrons was measured using a hemispherical energy analyzer at pass energies ranging from 50 to 150 eV. Data were collected at a photoelectron takeoff angle of 45 degrees with respect to the sample surface. The binding energy (BE) scale was referenced by setting the peak maximum in the C1s spectrum to 284.6 eV. The obtained high-resolution C1s spectra were fitted using Shirley background subtraction and a series of Gaussian peaks.

The storage modulus of the PVA-IA hydrogels prepared from a 5 wt% PVA-IA solution and crosslinked for 0.5–48 h were quantified using a rheometer (Physica MCR 101, Anton Pars Co. Ltd.) with a 5% strain at 1 Hz.

### hPSC culture

The hESC line WA09 (H9) was maintained as previously described on mitomycin-C-treated mouse embryonic fibroblast (MEF) feeder cells in DEME/F12 medium supplemented with 20% KnockOut Serum Replacement^56^. WA09 cells were split into two lines: one group of WA09 cells was maintained on MEFs and the other group was maintained on Matrigel as previously described^42^. The hiPSC line HS0077 was maintained on Matrigel in Essential 8 medium. Experimental control cells were obtained from conventional colony cultures and maintained as previously described^11^. Briefly, near-confluent cells were incubated with 2 mg/ml dispase in DMEM/F-12 medium at 37 °C for 2 min and then rinsed twice with DMEM/F12 medium. After addition of DMEM/F-12 medium, weakly adherent colonies were detached using a cell scraper. Cells were collected and centrifuged at 160 × g for 5 min at 37 °C. Small colonies were passaged into Matrigel-coated dishes or MEFs at a 1:4 ratio. Colonies showing morphological differentiation were manually removed under a microscope during each passage except at the time of analysis. Completely dissociated cells were cultured after preculture in the appropriate defined medium (mTeSR1 or Essential 8) for nine passages on Matrigel-coated dishes to remove MEF feeders. This stage was defined as passage 0. Near-confluent cell clusters were treated with dispase for 1–2 min at 37 °C. Cells in defined medium were pipetted to disperse the cells completely. After centrifugation at 160 × g for 5 min at 4 °C, the cells were seeded at the appropriate density (5x10^4^ cells per cm^2^ for passaging or as indicated) into new culture dishes (PVA-IA hydrogels grafted with oligoVN). The medium was changed daily for all experiments.

### hPSC characterization

The alkaline phosphatase (AP) activity of hiPSCs was measured using an alkaline phosphatase detection kit according to the manufacturer’s instructions^26^.

Immunostaining of Oct3/4, Sox2, SSEA-4, and Tra-1-81 was performed on hPSCs to evaluate pluripotency following the conventional protocol^26^. The stained cells were analyzed using fluorescence microscopy (Eclipse Ti-U fluorescence inverted microscope, Nikon Instruments, Inc., Tokyo, Japan).

### Embryoid body formation

hPSC pluripotency was evaluated by embryoid body (EB) formation at passages 10 and 20. hPSCs were dissociated from the substrate as described above for passages. Cells in the supernatant were collected, counted using a hemocytometer, and seeded onto ultra-low attachment dishes in Essential 6 medium to form EBs. After 2 weeks in suspension, EBs were transferred to Matrigel-coated dishes and cultured in Eseential 6 medium for an additional 3–5 weeks. Then, the cells were stained with antibodies against markers of all three embryonic germline layers (AFP, GFAP, βIII-Tubulin, and SMA) and analyzed by the immunofluorescence method^19^.

### Teratoma formation

The experiments in this study were approved by the ethics committees of the National Central University and the Taiwan Landseed Hospital (IRB-13-05). All experiments were performed in accordance with all applicable and relevant institutional and governmental regulations and guidelines during this study. Cells were harvested by treatment with a non-enzymatic cell dissociation solution. After centrifugation, the pellets were suspended in DMEM/F12-Matrigel. In total, 5 × 10^6^ cells were injected subcutaneously into SCID mice (6–8 weeks). After 6–8 weeks, teratomas were dissected and fixed with formaldehyde solution. Paraffin-embedded teratomas were sectioned and stained with hematoxylin and eosin^19^.

### Statistical analysis

All of the quantitative results were obtained from four samples. The data are expressed as the mean ± SD. Statistical analyses were performed using the unpaired Student’s *t*-test in Excel (Microsoft Corporation). Probability values (*p*) less than 0.05 were considered statistically significant.

## Additional Information

**How to cite this article**: Higuchi, A. *et al.* Long-term xeno-free culture of human pluripotent stem cells on hydrogels with optimal elasticity. *Sci. Rep.* **5**, 18136; doi: 10.1038/srep18136 (2015).

## Supplementary Material

Supplementary Information

## Figures and Tables

**Table 1:** Coating and substrates for feeder-free hPSC culture in a defined medium^a^.

(a)	Coating materials of ECMs
	Gelatin^19^, fibronectin^23^, laminin^18^, **laminin-332**^11^, **laminin-511**^12^, **laminin-521**^13^, **vitronectin**^15^, **recombinant vitronectin**^14^
(b)	Polysaccharide substrates
	Chitin/alginate, cellulose, positively charged cellulose
(c)	Oligopeptides for immobilization on substrates
	Poly-D-lysine, cyclicRGD^27^, pronectin^28^, **oligovitronectin**^25^,^26^
(d)	Chimera protein for immobilization on substrates
	E-cadherin chimera^53^
(e)	Synthetic polymer of substrates^b^
	MEASAH, PMVE-alt-MA^39^, PMEDSAH^40^,^41^,^42^,^43^, APMAAm^44^,Copoly(AEtMA-co-DEAEA)^45^, Poly-3,4-dihydroxy-L-phenyl-alanine

^a^Bold biomolecules are typically used.

^b^PMVE-alt-MA, poly(methyl vinyl ether-alt-maleic anhydride); PMEDSAH, poly[2-(methacryloyloxy(ethyl dimethyl-(3-sulfopropyl)ammoniumhydroxide; APMAAm, aminopropylmethacrylamide; Copoly(AEtMA-co-DEAEA), copoly[2-(acryloyloxyethyl) trimethylammonium-co-2-(diethylamino)ethyl acrylate].
