# Supplementary material for: Long-term xeno-free culture of human pluripotent stem cells on hydrogels with optimal elasticity
Source: Sci Rep. 2015 Dec 14;5:18136. doi: 10.1038/srep18136 (PMC4677349; doi:10.1038/srep18136)
Supplement: Supplementary Information [file srep18136-s1.pdf]

Supplementary Material for Long-term xeno-free culture of human pluripotent stem cells on hydrogels with optimal elasticity

Akon Higuchi[1,2,3,4], Shih-Hsuan Kao[1], Qing-Dong Ling[5,6], Yen-Ming Chen[1], Hsing-Fen Li [1], Abdullah A. Alarfaj[4], Murugan A. Munusamy[4], Kadarkarai Murugan[7], Shih-Chang Chang[8], Hsin-Chung Lee[8,9], Shih-Tien Hsu[10], S. Suresh Kumar[11], Akihiro Umezawa[2]

[1]Department of Chemical and Materials Engineering, National Central University, No. 300 Jhongli, Taoyuan, 32001 Taiwan

[2]Department of Reproduction, National Research Institute for Child Health and Development, 2-10-1 Okura, Setagaya-ku, Tokyo 157-8535, Japan

[3]Nano Medical Engineering Laboratory, RIKEN, 2-1, Hirosawa, Wako, Saitama 351-0198, Japan

[4]Department of Botany and Microbiology, College of Science, King Saud University, P.O. Box 2455, Riyadh 11451, Saudi Arabia

[5]Cathay Medical Research Institute, Cathay General Hospital, No. 32, Ln 160, Jian-Cheng Road, Hsi-Chi City, Taipei, 221, Taiwan

[6]Graduate Institute of Systems Biology and Bioinformatics, National Central University, No. 300, Jhongda RD., Jhongli, Taoyuan, 32001 Taiwan

[7]Division of Entomology, Department of Zoology, School of Life Sciences, Bharathiar University, Coimbatore, Tamil Nadu, India

[8]Department of Surgery, Cathay General Hospital, No.280, Sec. 4, Ren'ai Rd., Da'an Dist.,Taipei, 10693, Taiwan

[9]Graduate Institute of Translational and Interdisciplinary Medicine, College of Health Science and Technology, National Central University, No. 300, Jhongda RD., Jhongli, Taoyuan, 32001 Taiwan

[10]Department of Internal Medicine, Taiwan Landseed Hospital, 77, Kuangtai Road, Pingjen City, Taoyuan 32405, Taiwan

[11]Department of Medical Microbiology and Parasitology, Universities Putra Malaysia, Serdang 43400, Slangor, Malaysia

SUPPLEMENTARY FIGURES

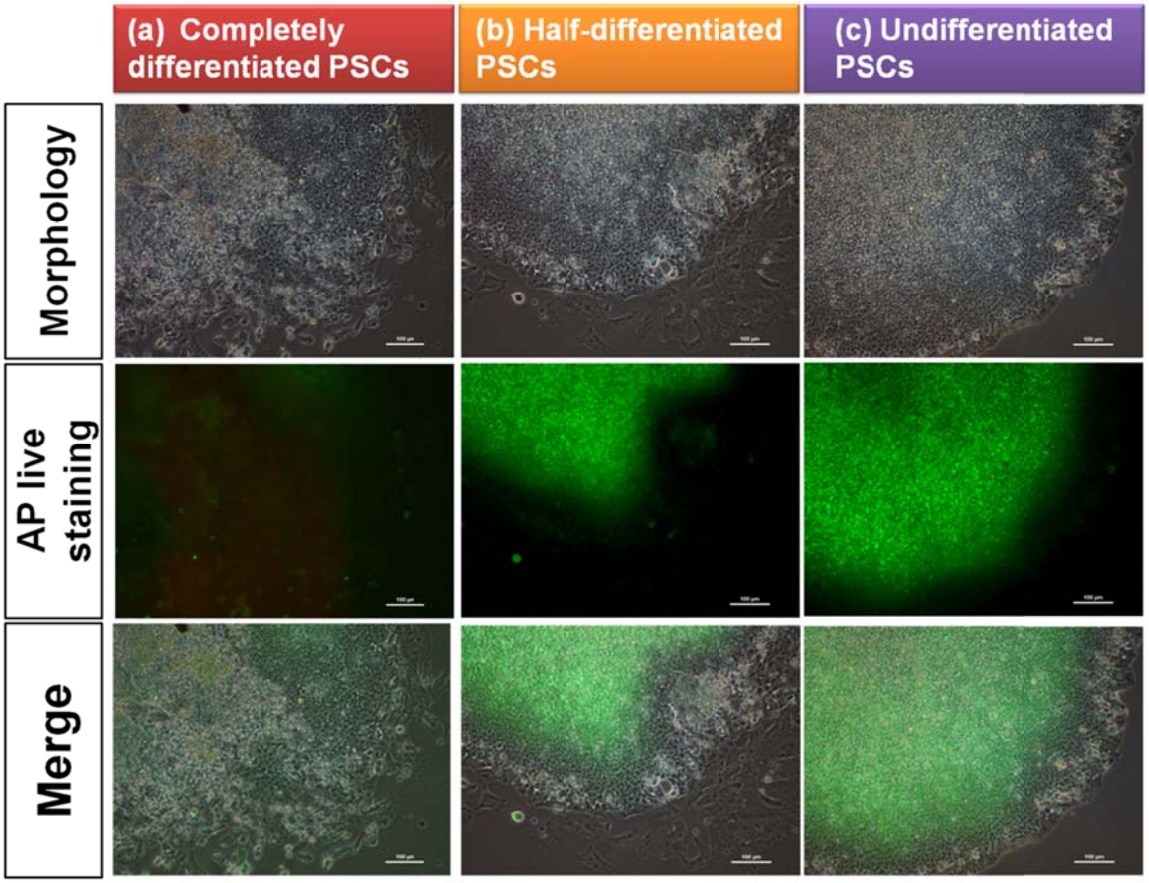

Supplementary Figure 1. Definition of completely differentiated PSCs (a), partially differentiated PSCs (b), and pluripotent (undifferentiated) PSCs (c). No alkali phosphatase (AP) activity was detected in the completely differentiated cells (a). In contrast, while the edge of the colony of the partially differentiated cells did not exhibit alkali phosphatase activity, alkali phosphatase activity was detected in the center of the colony (b). The pluripotent cells exhibited good colony morphology and alkali phosphatase activity in most cells (c).
